# Infectious Aortitis: A Life-Threatening Endovascular Complication of Nontyphoidal *Salmonella* Bacteremia

**DOI:** 10.1155/2018/6845617

**Published:** 2018-04-01

**Authors:** Seifeldin Hakim, Francisco Davila, Mitual Amin, Ismail Hader, Mitchell S. Cappell

**Affiliations:** ^1^Department of Medicine, Division of Gastroenterology, William Beaumont Hospital, Royal Oak, MI 48073, USA; ^2^Oakland University William Beaumont School of Medicine, Royal Oak, MI 48073, USA

## Abstract

A 65-year-old Japanese man living in the United States presented with pyrexia and chills associated with intermittent lower abdominal and back pain for 5 days. He denied recent travel, rash, diarrhea, or rectal bleeding. Physical examination revealed spiking pyrexia, and routine laboratory tests revealed mild leukocytosis and neutrophilia. Abdominal CT with contrast showed findings highly compatible with aortitis. Comprehensive autoimmune evaluation was negative. *Salmonella enterica* serotype Enteritidis was isolated from blood cultures. IV antibiotics were administered, but the patient continued to experience low-grade pyrexia and mild leukocytosis, and follow-up abdominal CT showed progressive aortic inflammation. The patient therefore underwent resection of the affected aortic segment with in-situ graft replacement and lifelong suppressive antibiotics. The patient is asymptomatic with no complications at 18 weeks of follow-up. This case report illustrates that patients with infectious aortitis from nontyphoidal *Salmonella* may (1) present with nonspecific and nonlocalizing symptoms and signs except for sepsis; (2) have diagnostic blood cultures and abdominal CT findings; and (3) typically require aggressive, prolonged IV antibiotic therapy and surgery for potential cure of this life-threatening infection.

## 1. Introduction

A case is reported of nontyphoidal *Salmonella* (NTS) causing aortitis and aneurysmal formation, which illustrates that patients with this infection can (1) present with nonspecific and nonlocalizing symptoms of sepsis; (2) have diagnostic blood cultures and abdominal CT findings, and (3) typically require aggressive, prolonged IV antibiotic therapy and surgery for potential cure of this life-threatening infection.

## 2. Case Presentation

A 65-year-old Japanese man with past medical history of hypertension and chronic hepatitis B, and no recent travel history, presented with diffuse body aches, spiking pyrexia, chills, intermittent lower back pain, and abdominal pain unrelated to meals for 5 days. He denied recent diarrhea, rash, or rectal bleeding. Physical examination revealed stable vital signs except temperature (38.9°C), no jaundice, soft nontender abdomen, guaiac-negative stools, no localized tenderness over the spine, and no limitation of spine mobility. Laboratory analysis revealed a leukocyte count of 10,100/*µ*L (normal: 3,500–10,100/*µ*L), neutrophil level of 7,400/*µ*L (normal: 1,600–7,200/*µ*L), hemoglobin level of 16.4 g/dL (normal: 13.5–17.0 g/dL), platelet count of 266,000/*µ*L (normal: 150,000–400,000/*µ*L), and serum sodium of 131 mmol/L (normal: 135–145 mmol/L), aspartate aminotransferase of 74 U/L (normal: 10–37 U/L), alanine aminotransferase of 81 U/L (normal: 9–47 U/L), and alkaline phosphatase of 101 U/L (normal: 30–110 U/L), with normal total bilirubin and albumin levels. Serum parameters of renal function were within normal limits. The erythrocyte sedimentation rate (ESR) was 107 mm/hr (normal: 0–15 mm/hr), and the C-reactive protein (CRP) level was 17.4 mg/dL (normal: 0–0.8 mg/dL). He was HIV seronegative, and rapid plasma reagin (RPR) was nonreactive. Comprehensive immunological evaluation was within normal limits, including levels/titers of immunoglobulin G subclasses, antinuclear antibodies, rheumatoid factor, cyclic citrullinated peptide, myeloperoxidase, proteinase 3 antibodies, and antineutrophylic cytoplasmic antibodies. The patient was given intravenous (IV) ciprofloxacin 400 mg twice daily empirically in the emergency room before admission.

Abdominal computerized tomography (CT) with intravenous contrast showed inflammatory changes of infrarenal aorta consistent with aortitis. *Salmonella enterica* serotype Enteritidis was isolated from multiple blood cultures. The bacterial isolate was susceptible to ampicillin, ceftriaxone, and trimethoprim/sulfamethoxazole, but not ciprofloxacin using automated sensitivity testing for 29 antibiotics (NM45, Beckman Coulter Labs., 250 S. Kramer Blvd., Brea, CA 92821).

**Figure 1:**
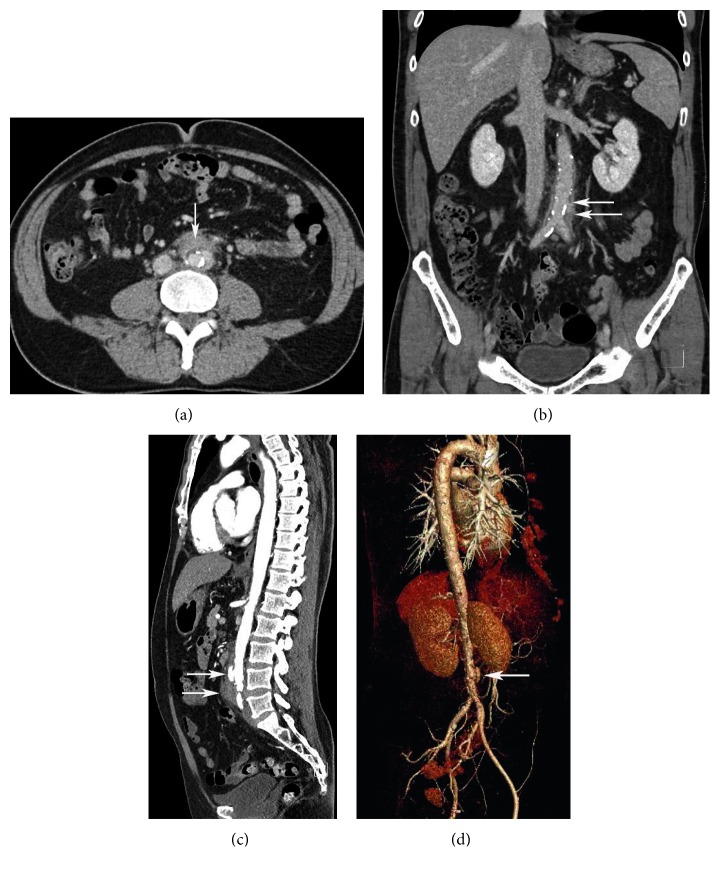
(a) Transverse view of abdominal CT shows periaortic soft tissue thickening adjacent to aorta (arrow). (b) Coronal view of abdominal CT without IV contrast shows disruption of calcification in aortic wall (upper arrow) and periaortic soft tissue thickening around the infrarenal aorta and around bifurcation of aorta into common iliac arteries (lower arrow). (c) Sagittal view of abdominal CT, with IV contrast, shows pseudoaneurysm formation (upper arrow) and periaortic soft tissue thickening around the infrarenal aorta (lower arrow). (d) Reconstructive CT angiogram shows pseudoaneurysm formation (arrow).

**Figure 2:**
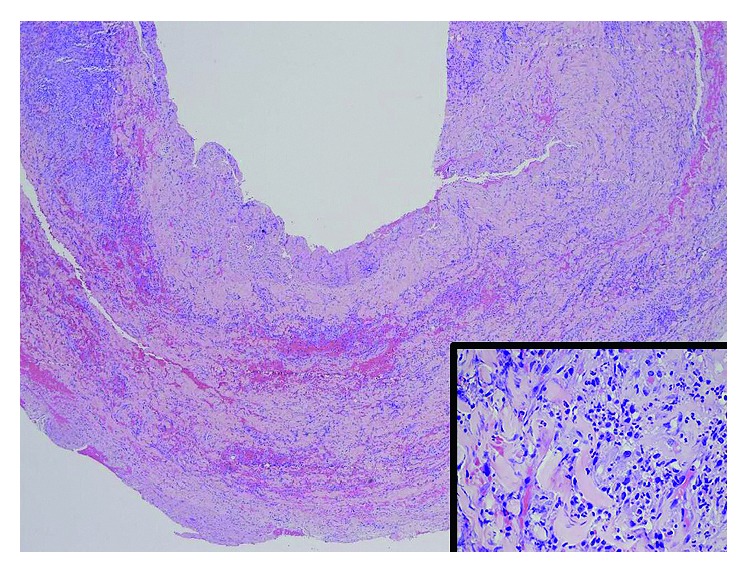
Low-power photomicrograph of resected aortic segment with hematoxylin and eosin stain shows inflammation of aortic wall. Inset: high-power photomicrograph of resected aortic segment with hematoxylin and eosin stain shows abundant neutrophylic infiltration of aortic wall without detection of the microorganism.

The antibiotic regimen was switched to IV ceftriaxone 2 g/24 hr, but the patient developed progressive abdominal and back pain and persistent pyrexia and mild leukocytosis. Follow-up abdominal CT one week later showed progressive inflammatory changes around infrarenal aorta extending to common iliac arteries and proximal external and internal iliac arteries, mild mural thickening of proximal abdominal aorta, and development of penetrating intimal ulcers, suggesting progression of aortitis (Figures 1(a)–1(d)). He underwent surgical resection of the infected aortic segment with in-situ aortoiliac rifampin-impregnated Dacron graft supported with bilateral iliac arterial stents. Histopathology of the resected aorta revealed severe neutrophylic infiltration and other inflammatory cells within the arterial wall, without evident bacteria (Figure 2). He was discharged after 1 week of IV ceftriaxone therapy to complete a further 5 week course of IV therapy as an ambulatory patient. The patient's CRP and ESR were followed weekly after admission. The CRP declined after 1 week to <0.4 mg/dL and consistently remained at this low level thereafter. The ESR declined progressively by about 20 mm/hr every week until reaching a steady-state level of about 22 mm/hr after 4 weeks. He is asymptomatic at 18-weeks follow-up with planned lifelong antibiotic suppressive therapy with amoxicillin.

## 3. Discussion

Infectious aortitis is a rare, life-threatening, inflammatory process within the aortic wall caused by microorganisms that can lead to aortic aneurysm and rupture [1]. 2.6% of abdominal aortic aneurysms are secondary to infectious aortitis [2]. Any microorganisms, such as bacteria or fungi, can cause infectious aortitis. In the pre-antibiotic era, syphilis and bacterial endocarditis were the most common infectious etiologies. Currently, common infectious organisms include Gram-positive bacteria such as *Staphylococcal* species, *Enterococcus*, or *Streptococcus* or Gram-negative bacteria such as *Salmonella*. Uncommon organisms in developed countries are *Mycobacterium tuberculosis* and *Treponema pallidum*, but the latter organisms are more common in developing countries. Fungi, such as *Candida* and *Aspergillus,* can also infect the aorta [3]. In general, healthy aortic intima is highly resistant to infection, and the major risk factor for aortic infection is atherosclerosis. Mechanisms of aortic infection include (1) most commonly, bacterial seeding of atherosclerotic plaques, (2) extension from contiguous infection, (3) penetrating trauma with direct microorganism invasion of aorta, (4) closed trauma with disruption of aortic intima which may facilitate seeding with microorganisms, (5) septic emboli of aortic vasa vasorum, and (6) self-inflicted or iatrogenic vascular manipulation [4, 5].

*Salmonella* is a Gram-negative, facultative anaerobic, motile, non-lactose-fermenting, and non-spore-forming bacillus [6]. NTS are ubiquitous in nature and infect poultry and reptiles [5]. *Salmonella* infection can lead to (1) gastroenteritis, (2) enteric fever, (3) bacteremia, (4) extraintestinal focal infection (EFI), (5) chronic carrier state, and (6) occasionally as bacteremia without gastrointestinal involvement that may lead to EFIs [5, 7]. *Salmonella* aortitis comprises about 40% of all infective aortitis [8, 9]. NTS, especially *Salmonella choleraesuis* and *typhimurium,* comprise one-third of abdominal aortic infections [3]. NTS aortitis has a predilection to infect the abdominal aorta, especially infrarenal segment [2]. Bacterial and leukocytic enzymes can further weaken the elastic fibers of the damaged aortic wall leading to aneurysmal formation [3].

Approximately 5% of patients with NTS gastroenteritis develop bacteremia [10]. EFIs of NTS can infect endovascular tissue, bone, brain, meninges, lungs, or abdominal viscera [10]. The incidence of EFI in NTS bacteremia is 40% [7]. Endovascular infection is a life-threatening extraintestinal complication of NTS bacteremia. About 25% of patients >50 years old with NTS bacteremia develop endovascular infections. Risk factors for vascular infection in a study of 358 patients >50 years old with NTS bacteremia included male sex, hypertension, coronary artery disease, and serogroup C1 infections, whereas negative risk factors include malignancy and immunosuppressive therapy [11]. Although immunosuppression predisposes subjects to NTS bacteremia, immunosuppression may counteract formation of endovascular infections [12].

Symptoms are variable, but may include the following: (1) gastroenteritis: diarrhea, nausea, vomiting, and abdominal cramps; (2) infectious symptoms: pyrexia, chills, and sweats; (3) aortitis: location of the involved aortic segment determines the type and severity of symptoms: abdominal pain if abdominal aorta is affected; chest, shoulder, or back pain if thoracic aorta is affected; and shock or hemodynamic instability if an aneurysm forms and ruptures; and (4) other symptoms of EFIs: endocarditis, septic arthritis, osteomyelitis, cholangitis, meningitis, pneumonia, or other manifestations of visceral organ involvement [2, 10].

Diagnosis of *Salmonella* aortitis requires a high index of suspicion in the presence of risk factors, NTS bacteremia, and compatible symptoms even in hemodynamically stable patients. Chen et al. [11] reported in 2012 a scoring system for NTS vascular infections (NTSVIs) to help predict the risk of vascular infections in patients with NTS bacteremia. In this scoring system, each of the following is assigned 1 point: male sex, hypertension, coronary artery disease, and serogroup C1 infection; and each of the following is assigned −1 point: malignancy, and immunosuppression due to negative association with aortitis. Based on this scoring system, the prevalence of vascular infections in patients with 0, 1, 2, 3, or 4 points was 2.2%, 10.6%, 39.4%, 55.2%, and 100%, respectively [11, 12].

Supporting data include laboratory studies showing leukocytosis and neutrophilia, elevated markers of inflammation such as ESR or CRP, and positive blood cultures [2, 7]. Pretreatment with antibiotics can lead to absence of microorganisms in the resected aortic specimen, as occurred in this case, and sterile blood cultures [4, 13].

Abdominal CT with IV contrast is the diagnostic study of choice. However, CT can show normal aorta during early aortitis, as it may miss the initial changes in the arterial wall or periaortic tissue [14]. Diagnostic CT findings of aortic inflammation include mural thickening, periaortic soft tissue density, rim enhancement, periaortic gas, periaortic stranding or fluid retention, saccular or fusiform aneurysm, disruption of calcification, and vertebral body erosion [15, 16]. Magnetic resonance imaging (MRI) with gadolinium enhancement is an emerging imaging technique that may have similar diagnostic potential as CT, but should not be used in patients with implanted devices or unstable patients [4]. Digital subtraction angiography (DSA) and invasive aortography are reserved for cases in which the diagnosis of acute aortitis cannot be excluded by noninvasive methods or to confirm the diagnosis. DSA and aortography have the disadvantage of imaging the aortic lumen only without showing adjacent soft tissue or bony involvement. Invasive aortography carries the additional risk of rupture of the fragile aortic wall from increased intraprocedural aortic pressure [4, 15].

NTS aortitis is usually treated by combined medical and surgical therapy. The mortality is 96–100% with medical therapy alone and is 40% with combined medical and surgical therapy [2]. Initial empiric antibiotic therapy for infectious aortitis should generally cover Gram-positive and Gram-negative bacteria, taking into consideration risk factors for multidrug-resistant organisms and/or extended spectrum beta-lactamase-resistant bacteria that may require specific antibiotics (e.g., vancomycin, ceftaroline, daptomycin, linezolid, carbapenem, or newer agents) [17, 18]. When *Salmonella* aortitis is confirmed, beta-lactam antibiotics (ceftriaxone, piperacillin-tazobactam, or other beta-lactams) are preferred if the bacteria are susceptible to them because some emerging *Salmonella* strains, especially in Asia, have increasing resistance to ciprofloxacin and other conventional antibiotic therapies [19]. If surgery is not urgently indicated (neither impending aortic rupture nor hemodynamic instability), intravenous antibiotics should be administered for 2–4 weeks before surgery to control the local infection to prevent reinfection, especially for in-situ graft placement. Intravenous antibiotics should be extended postoperatively until 6–12 weeks after blood cultures become sterile [2, 13].

The optimal duration of antibiotic therapy remains controversial, with some experts suggesting lifelong suppressive oral antibiotic therapy after IV antibiotics especially for difficult to treat microorganisms or for in-situ graft placement [20]. Long-term suppressive antibiotic therapy with oral agents depends on culture susceptibility, but the data are limited about specific antibiotics, antibiotic dosages, and duration of therapy [21–23]. Most studies of antibiotic therapies included ciprofloxacin, amoxicillin, amoxicillin-clavulanic acid, or trimethoprim-sulfamethoxazole antibiotic agents.

The standard surgical treatment is resection of the infected aortic segment and in-situ or extra-anatomical reconstruction. Theoretically, in-situ graft is more prone to graft reinfection, whereas extra-anatomical graft is more prone to thrombosis [4]. Endovascular aortic repair (EVAR) can be used as a temporary measure to hemodynamically stabilize unstable patients or to seal vascular leaks until the patient undergoes definitive surgery. EVAR has been used as a stand-alone technique to surgically repair mycotic aortic aneurysms and *Salmonella* aortic aneurysms, but the current data on efficacy are inadequate [3, 4, 10, 23, 24]. Study limitations include this is a single case report, and it is retrospectively reported.

## 4. Conclusion

A case is reported of NTS causing aortitis and aneurysmal formation. The patient presented nonspecifically with diffuse body aches, spiking pyrexia, chills, intermittent lower back pain, and abdominal pain unrelated to meals for 5 days, without recent diarrhea, rash, or rectal bleeding. Blood cultures and abdominal CT findings were diagnostic. The patient was successfully treated with prolonged IV antibiotic therapy, segmental aortic resection, and graft repair. This case illustrates the potential elusiveness of this diagnosis.

NTS aortitis is a life-threatening disease associated with up to 100% mortality if inadequately treated. Early diagnosis is crucial to improve outcome. However, the diagnosis can be challenging as blood cultures can be sterile due to prior antibiotic use, CT scan can be normal early in the infection, and other diseases can lead to aortitis. Diagnosis of NTS aortitis requires awareness of risk factors, recognition of symptoms, and a high index of suspicion. CT scan is the imaging modality of choice. Early diagnosis, early use of appropriate antibiotics, and definitive surgical repair may reduce mortality to 40%.
